# Detection and Control of Fungal Outbreaks

**DOI:** 10.1007/s11046-020-00494-1

**Published:** 2020-10-10

**Authors:** Diego H. Caceres, Ratna Mohd Tap, Ana Alastruey-Izquierdo, Ferry Hagen

**Affiliations:** 1 Mycotic Diseases Branch, Centers for Disease Control and Prevention (CDC), 1600 Clifton Rd. NE, Atlanta, GA 30329 USA; 2 Center of Expertise in Mycology Radboudumc/CWZ, Nijmegen, The Netherlands; 3 Mycology Laboratory, Institute for Medical Research, National Institute of Health, Setia Alam, 40170 Shah Alam, Selangor Malaysia; 4 Medical Mycology Reference Laboratory, National Center for Microbiology, Instituto de Salud Carlos III, Madrid, Spain; 5 Department of Medical Mycology, Westerdijk Fungal Biodiversity Institute, Uppsalalaan 8, 3584CT Utrecht, The Netherlands; 6 Department of Medical Microbiology, University Medical Center Utrecht, Heidelberglaan 100, 3584 CX Utrecht, The Netherlands; 7 Laboratory of Medical Mycology, Jining No. 1 People’s Hospital, Jining, Shandong People’s Republic of China

**Keywords:** Emerging pathogen, Fungal infection, Immunodiagnostics, Molecular diagnostics, Molecular epidemiology, Nosocomial outbreak

The fungal kingdom contains an estimated 2.2–3.8 million species [1]. Fungi are ubiquitous and primarily co-exist with plants and animals, creating mutual benefits, including the acquisition of vital nutrients and protection from pathogenic microorganisms. Generally, fungi are beneficial to humankind and are indispensable in the food production chain and in biotechnological processes like the production of chemical compounds and drugs. Approximately 600 species have been reported to cause human disease, mostly due to traumatic introduction into the host. An even smaller number cause serious superficial or invasive disease (https://www.clinicalfungi.org/). The dermatophytes cause superficial infections, but also deep infections, and negatively impact the quality of life of ~ 1 billion people worldwide [2–5]. Globally, *Aspergillus*, *Candida*, *Cryptococcus*, *Pneumocystis*, and Mucorales infections (the ‘big five’) are important causes of fungal morbidity and mortality, primarily affecting immunocompromised hosts [2, 6, 7]. Among these big five, *Candida* yeasts cause the largest number of infections, with annual estimates of 134 million mucosal infections, as well as 750,000 life-threatening bloodstream infections associated with mortality rates > 40% [2, 8].

Outbreaks caused by fungi can be classified into two groups. The first consists of community-acquired outbreaks that are linked to outdoor activities, mostly seen in patients who perform a specific occupational or recreational activity and those who experienced a natural disaster or human conflicts [9–12]. The second group of fungal outbreaks is related to hospital care and has been increasingly reported during the past two decades, likely as a result of the increase in at-risk populations [13]. The sources of healthcare-associated outbreaks include construction near or renovation of healthcare facilities, contamination of water and ventilation systems, medicines and medical devices, and gaps in infection prevention measures [9–12, 14, 15]. An increasingly reported cause of fungal outbreaks is the emergence of novel resistant pathogens within hospital environments, clearly exemplified by the unprecedented emergence of *Candida auris*, as well as increasing incidence of previously rare resistant species like *Candida krusei* and *Diutina rugosa* [16–19].

This special issue covers several aspects of fungal outbreaks. The use of immunodiagnostic and molecular tests for the detection and control of community and healthcare-associated fungal outbreaks is presented [20] and specifically reviewed for Mucorales and the unculturable *Pneumocystis jirovecii* [6, 7]. Fungal immunodiagnostic assays can detect either antigens, antibodies or metabolites, and are available in different methodologies, presenting from high laboratory complex complement fixation, to the point-of-care lateral flow assays. These assays are developed to detect specific or multiple pathogens. Caceres and colleagues reviewed the available immunodiagnostic assays and provided the ins and outs of these assays as well as an overview of the strengths and weaknesses of using each of them in a potential outbreak setting [20]. Linder and colleagues evaluated the performance of the ‘pan-fungal’ (1-3)-β-d-glucan (BDG) assay for the diagnosis of invasive pulmonary aspergillosis (IPA) using bronchoalveolar lavage (BAL) samples; they concluded that diagnostic accuracy of BDG using BAL was poor for the diagnosis of IPA [21]. Mucorales is an illustrious yet enigmatic cause of opportunistic infections and outbreaks. Despite their rapid progressive growth, they are difficult to diagnose as they cannot be detected by an immunodiagnostic assay, like the BDG assay, and thus culture (often negative), histopathology, imaging, and molecular diagnostics remain the diagnostic arsenal from which to choose. Walther and co-workers identified 32 outbreaks of mucormycosis, the majority (*n* = 28) were hospital-acquired and four were community-acquired [7]. Interestingly, Walther and colleagues observed that only in few outbreaks molecular typing was applied to investigate clonality (see Table 1 in [7]). Hence, we can conclude that in the case of a suspected Mucorales outbreak there is much more to explore from a molecular epidemiological point of view. While sensitivity of culture in Mucorales is very low, *Pneumocystis* species are non-culturable fungi [22, 23]; thus, molecular tools are essential to diagnose and study the epidemiology of this notorious culprit of nosocomial fungal outbreaks [6].

Two other fungal-like pathogens that are reviewed in this special issue are *Prototheca* [24] and *Pythium* [25], rarely identified by routine diagnostics but known to cause outbreaks. *Prototheca* are achlorophyllic algae that have a yeast-like colony morphology and therefore historically been studied by medical mycologists [24, 26]. Despite a recent outbreak among humans, protothecosis remain a relative rare cause of human disease, while it is widely known among veterinarians as a cause of bovine mastitis. Kano provided a summary on the history, biology, diagnostics, treatment, and current knowledge of *Prototheca* [24]. Like *Prototheca*, the aquatic oomycete *Pythium insidiosum* has been historically classified as a fungus, while it seems to be closer related to diatoms and algae [25, 26]. In their review, Permpalung and colleagues provided an overview of the diagnosis and treatment of this quirky pathogen that causes infections—ranging from keratitis to life-threatening—in both humans and animals [25].

Infections with *Talaromyces marneffei* are geographically restricted to southeast Asia and are seen predominantly in people living with HIV [27]. Six decades ago, *Penicillium marneffei* was described as a novel pathogen, but since several years it is grouped as the sole pathogenic and dimorphic species within the genus *Talaromyces* [28, 29]. The history of *T. marneffei* was recently extensively reviewed [27], but it deserves attention in this special issue as Sethuraman and co-workers reported five apparent autochthonous cases among patients with different underlying disorders that lived outside the endemic region [30]. This highlights that the current map of endemic mycoses needs to be redrawn, not only for *T. marneffei* but also for *Blastomyces*, *Coccidioides*, *Histoplasma*, and *Paracoccidioides* as their incidence and geographic range has changed [31]. Ashraf and colleagues included an overview of reported cases due *Emergomyces* species, and they stated that the epidemiology is incomplete and that our understanding continues to evolve which is depicted by a recent reported case of *E. pasteurianus* from India [32].

Molecular typing is essential to understand emergence and spread of novel fungal pathogens. An overview of epidemiological typing tools, ranging from microsatellite typing, multi-locus sequencing typing to whole genome sequencing, for *Candida glabrata* provided alternatives to track and control the spread of this yeast [33]. A molecular diagnostic approach was used to study the natural reservoirs of *Histoplasma capsulatum* by investigating the presence of the pathogen in stored Argentinian wildlife tissue samples [34]. An in-depth literature study spanning seven decades of epidemiological data from Brazil identified characteristics of histoplasmosis of outbreaks on this country. Based on this characterization, authors suggested interventions to prevent exposure [35]. Rodrigues and co-workers reviewed the global epidemiology of *Sporothrix* species is in all aspects, and refined maps for the three major pathogenic species *S. brasiliensis, S. schenckii*, and *S. globosa* [36].

The detection and control of fungal outbreaks is complex and require coordination between the clinical microbiology laboratory, clinicians, epidemiologists, and public health microbiologists, including other professionals (environmental and veterinary staff) to ensure a ‘One Health’ approach. These professionals must form an investigative multidisciplinary team, together with decision-makers to ensure implementation of control measures [37]. The global lack of awareness of fungal diseases, limited laboratory capacity, and the absence of fungal diseases on the radar of most epidemiological and public health systems are major challenges [38]. The World Health Organization (WHO) and its regional offices, the Mycotic Disease Branch of the Centers for Disease Control and Prevention (CDC), the Global Action Fund for Fungal Infections (GAFFI), Leading International Fungal Education (LIFE), International Society for Human and Animal Mycology (ISHAM), Mycoses Study Group Education and Research Consortium (MSG-ERC), European Society for Clinical Microbiology & Infectious Diseases—Fungal Infections Study Group (ESCMID/EFISG), and the European Confederation of Medical Mycology (ECMM) have launched a variety of initiatives, focused principally in education and advocacy of fungal diseases. These initiatives range from the Fungal Disease Awareness Week (September 21–25, 2020) to e-courses, specialized working groups, and guideline development [39–44]. Given the increasing global emergence of novel multidrug-resistant outbreak-causing fungal pathogens, coordinated international interdisciplinary collaboration to manage them is needed more than ever.
